# Charge-transfer interactions between fullerenes and a mesoporous tetrathiafulvalene-based metal–organic framework

**DOI:** 10.3762/bjnano.10.183

**Published:** 2019-09-18

**Authors:** Manuel Souto, Joaquín Calbo, Samuel Mañas-Valero, Aron Walsh, Guillermo Mínguez Espallargas

**Affiliations:** 1 Instituto de Ciencia Molecular (ICMol), Universidad de Valencia, C/ Catedrático José Beltrán 2, 46980 Paterna, Spain; 2 Department of Materials, Imperial College London, London SW7 2AZ, United Kingdom

**Keywords:** charge transfer, donor–acceptor, fullerene, metal–organic frameworks (MOFs), tetrathiafulvalene (TTF)

## Abstract

The design of metal–organic frameworks (MOFs) incorporating electroactive guest molecules in the pores has become a subject of great interest in order to obtain additional electrical functionalities within the framework while maintaining porosity. Understanding the charge-transfer (CT) process between the framework and the guest molecules is a crucial step towards the design of new electroactive MOFs. Herein, we present the encapsulation of fullerenes (C_60_) in a mesoporous tetrathiafulvalene (TTF)-based MOF. The CT process between the electron-acceptor C_60_ guest and the electron-donor TTF ligand is studied in detail by means of different spectroscopic techniques and density functional theory (DFT) calculations. Importantly, gas sorption measurements demonstrate that sorption capacity is maintained after encapsulation of fullerenes, whereas the electrical conductivity is increased by two orders of magnitude due to the CT interactions between C_60_ and the TTF-based framework.

## Introduction

Metal–organic frameworks (MOFs), which are crystalline porous materials constructed from metallic nodes and organic linkers, have been a major breakthrough in chemistry in the last decades [1–2]. Because of their immense structural and functional possibilities, this class of hybrid materials finds several applications in, for example, gas storage and separation, sensing or catalysis [3–5]. In addition, electroactive MOFs combining porosity and electrical conductivity [6–8] have also attracted much attention during the last years in view of their potential application, for example as chemiresistive sensors [9], field-effect transistors [10] or supercapacitors [11]. Whereas most MOFs are electrical insulators, some have shown to exhibit excellent electrical conductivity and high charge mobility. This was achieved either by an appropriate choice of the building units to form electronically delocalised frameworks, or by incorporating electroactive guest molecules in the pores [6,12–14]. In this direction, the incorporation of redox-active moieties [15–18] as well as the understanding of charge-transfer (CT) processes in MOFs [19–24], are excellent pathways for the rational design of new electroactive frameworks exhibiting electrical conductivity and porosity at the same time.

Fullerenes (C_60_) [25] have found numerous applications in different fields, ranging from molecular electronics and nanotechnology to biomedical applications, due to their exceptional electrochemical and photophysical properties [26–27]. In particular, understanding the CT processes between the electron-acceptor C_60_ and the electron-donor molecules is fundamental in order to optimise photovoltaics and develop efficient solar cells [28]. The encapsulation of C_60_ in MOFs [29] has become a very interesting strategy for the purification of fullerenes [30–32], or to incorporate additional functionalities within the MOF [33–35]. Very recently, Farha and co-workers have demonstrated that encapsulation of C_60_ in a zirconium-based MOF can lead to an enhancement of electrical conductivity due to donor–acceptor interactions between the pyrene-based ligand (donor) and fullerene (acceptor) without a significant decrease in the porosity [36].

Tetrathiafulvalene (TTF) and its numerous derivatives are redox-active electron-donor molecules with unique electronic properties that have been widely used as important building units in the field of molecular electronics as conductors, switches, sensors or rectifiers [37–38]. Several studies have also been devoted to the development of TTF-based macrocyclic systems for their use as molecular machines or for supramolecular host–guest recognition [39–41]. In this context, donor–acceptor interactions between C_60_ and discrete π-extended TTF molecules have been extensively studied in solution during the last years [42–47]. In contrast, much less is known about their supramolecular interactions in solid-state polymeric structures such as metal–organic frameworks.

**Figure 1:**
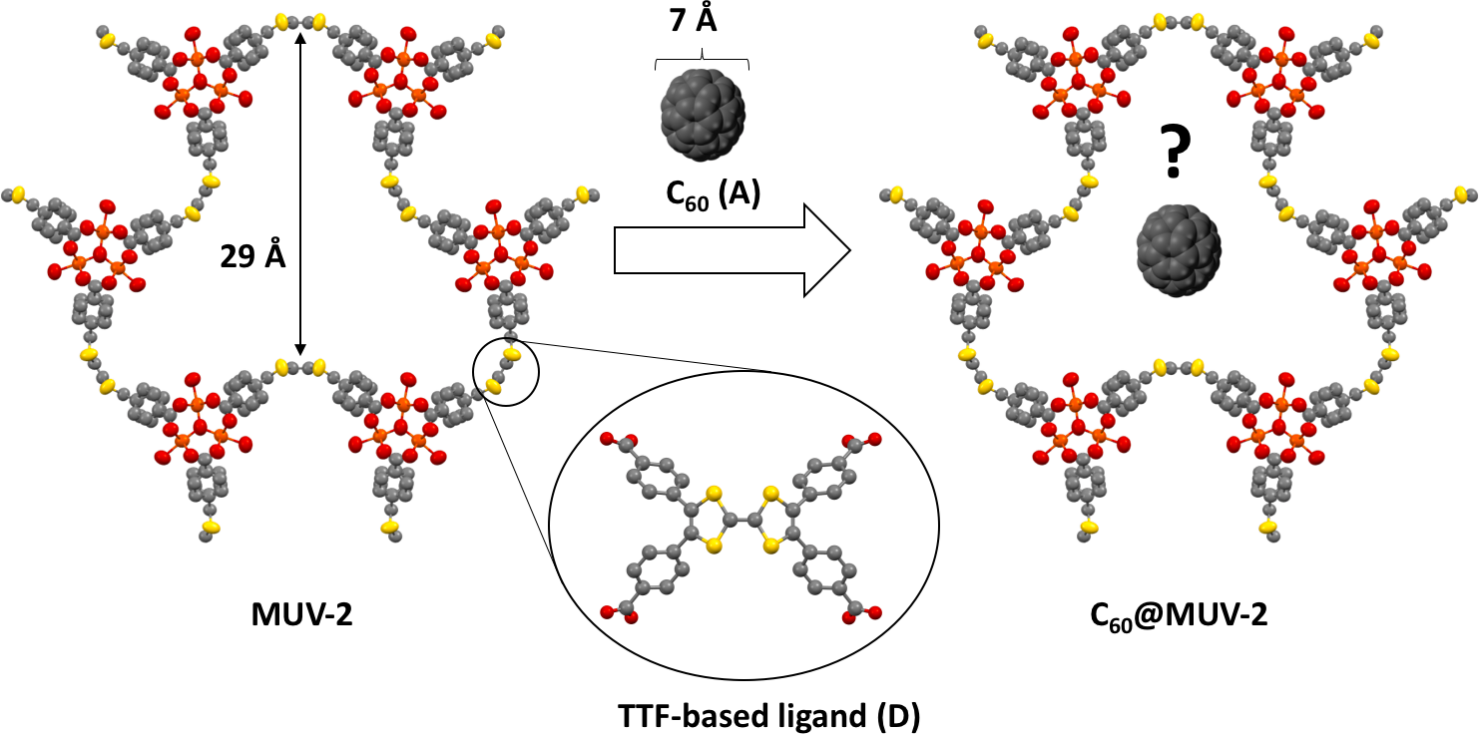
Schematic representation of the encapsulation of the electron-acceptor (A) C_60_ in the electron-donor (D) TTF-based **MUV-2**. Grey, yellow, orange and red spheres represent C, S, Fe and O atoms, respectively.

MOFs using TTF as ligands have become an interesting new class of functional porous systems since they can incorporate additional electronic features to prepare new electrically conductive and redox-active MOFs [48–51]. Very recently, we have reported a hierarchical and highly stable TTF-based MOF, named **MUV-2**, which is based on the 6-connected trimeric cluster [Fe_3_(μ_3_O)(COO)_6_] as secondary building unit (SBU) and tetratopic tetrathiafulvalene-tetrabenzoate (TTFTB^4−^) ligands. This MOF shows a hierarchical structure with mesoporous channels of ≈3 nm and orthogonal microporous channels of ≈1 nm. In addition, it exhibits an enhanced catalytic activity for the aerobic oxidation of dibenzothiophene in diesel [52], and a reversible continuous breathing upon adsorption of different solvents [53]. Importantly, the planarity of the TTF ligands can be modulated by the breathing behaviour, which directly impacts on its electrochemical properties [53–54]. In view of the remarkable electron-donor character of the TTF-based ligands, herein we present the encapsulation of C_60_ in **MUV-2** (**C_60_@MUV-2**) (Figure 1). A detailed study on the CT interactions between the electron-donor TTF ligands from the framework and the electron-acceptor fullerenes has been carried out by different spectroscopic techniques and theoretical calculations. Gas sorption measurements demonstrate that permanent porosity is retained, whereas electrical measurements show that conductivity is enhanced after C_60_ encapsulation.

## Results and Discussion

### Synthesis and characterisation of C_60_@MUV-2

#### Synthesis and encapsulation of C_60_ into MUV-2

**Figure 2:**
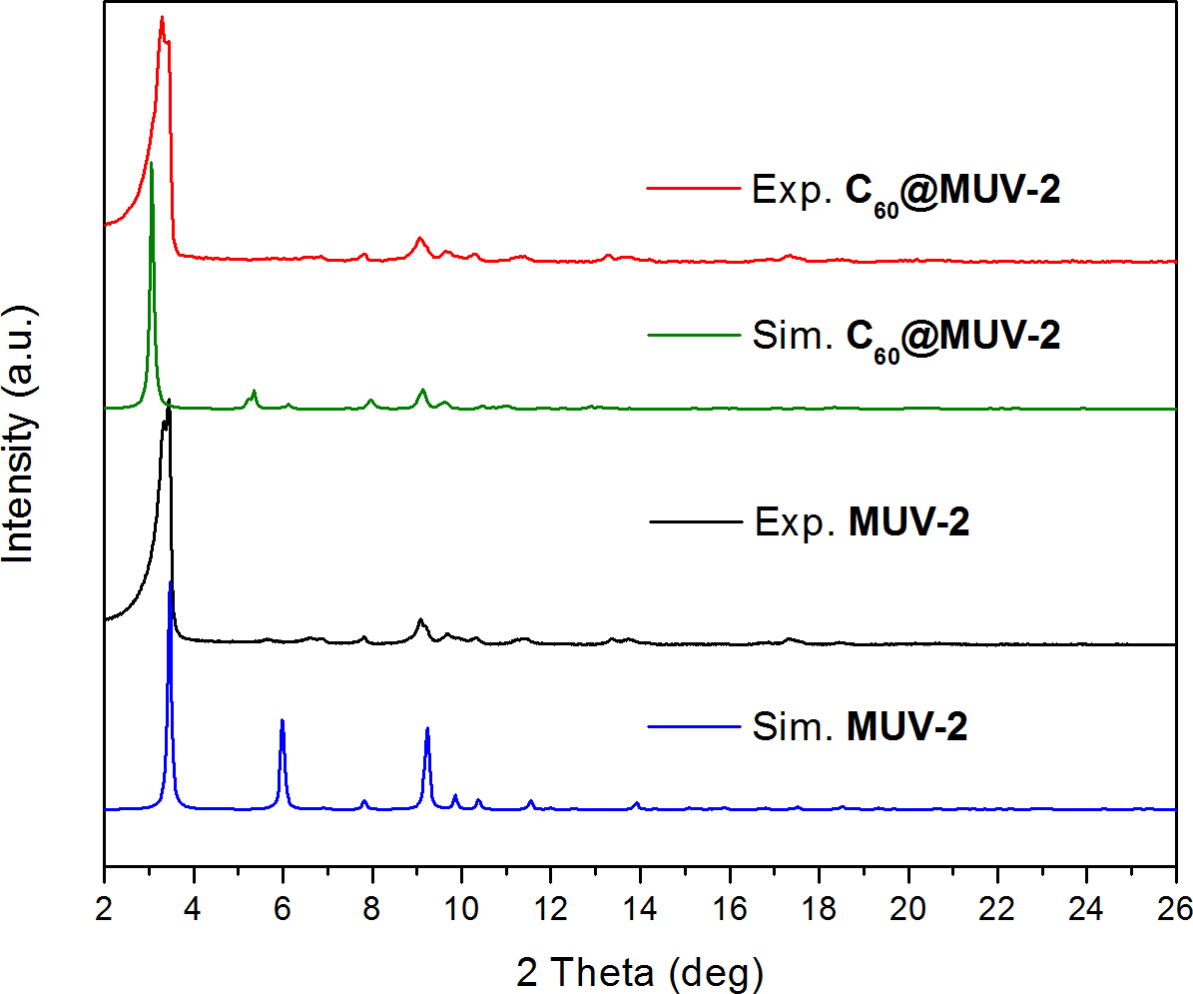
Powder X-ray diffraction (PXRD) patterns of simulated and experimental desolvated **MUV-2** and **C_60_@MUV-2**.

**MUV-2** was synthesised as previously described based on the solvothermal reaction of tetrathiafulvalene tetrabenzoic acid (H_4_TTFTB), the preformed cluster [Fe_3_O(CH_3_COO)_6_]ClO_4_ and acetic acid as a modulator in dimethylformamide (DMF) [52]. In order to activate the material, **MUV-2** was exhaustively washed with DMF, methanol and heated at 150 °C for 2 h. Encapsulation of C_60_ was achieved adapting a reported procedure [36] by immersing the activated microcrystalline powder of **MUV-2** in a saturated solution of C_60_ in *o*-dichlorobenzene for three days at 60 °C. Then, the material was exhaustively washed with *o*-dichlorobenzene in order to remove the physisorbed C_60_ on the MOF surface, washed with methanol and dried at 150 °C for 2 h. The powder X-ray diffraction (PXRD) pattern of **C_60_@MUV-2** shows that the principal peak remains at 3.4° confirming that crystallinity is maintained after encapsulation of C_60_ and removal of the solvent (Figure 2). It is important to note that the principal peak is slightly shifted when comparing the experimental and simulated PXRD patterns. This can be explained by the breathing behaviour of **MUV-2** [53]. The needle-like morphology of **C_60_@MUV-2** also remained similar to the one of **MUV-2** as confirmed by scanning electron microscopy (SEM) (Figure S1, Supporting Information File 1).

#### Raman and UV–vis spectroscopy

**Figure 3:**
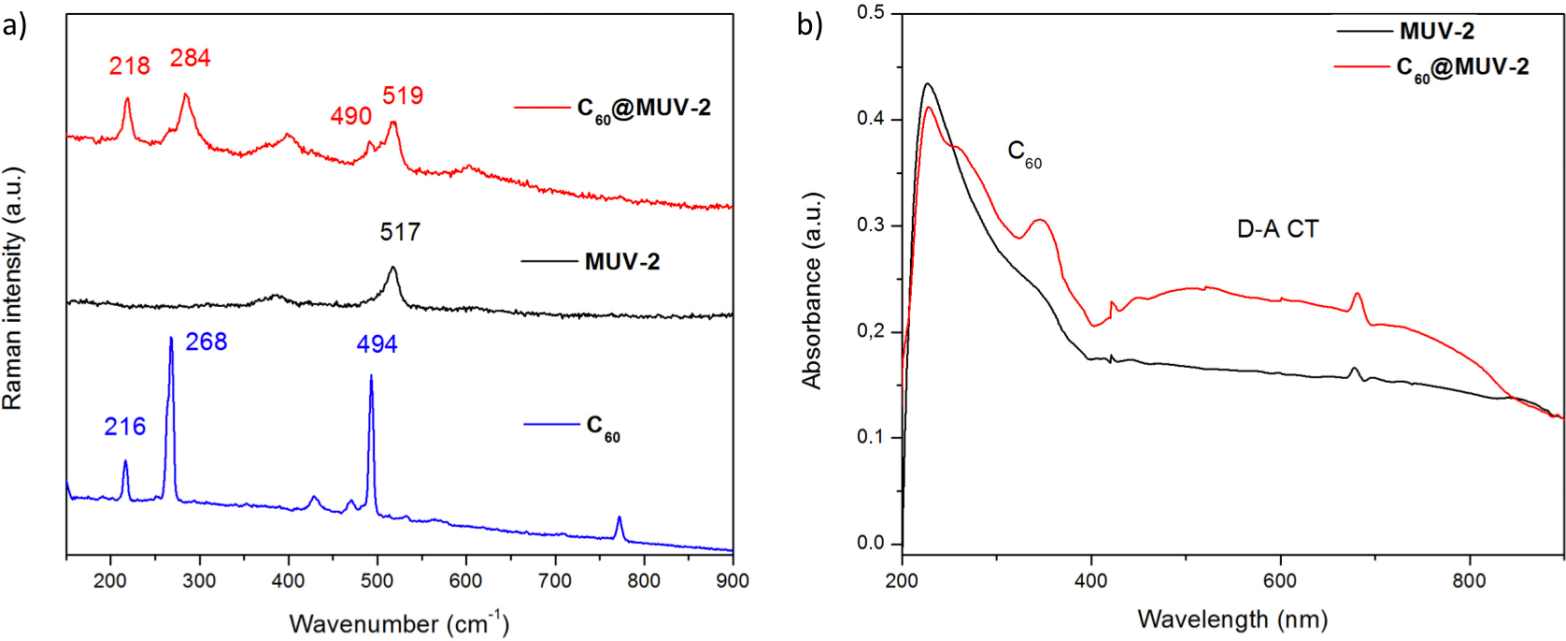
a) Raman spectra of **C_60_**, **MUV-2** and **C_60_@MUV-2**. b) Solid-state UV–vis spectra of **MUV-2** and **C_60_@MUV-2**. The spectra were recorded by dispersing the samples in KBr pellets.

Raman spectra of **C_60_**, **MUV-2** and **C_60_@MUV-2** crystals were measured using a Raman excitation wavelength of 785 nm (Figure 3a). The presence of Raman bands at 218, 284 and 490 cm^−1^ evidences the encapsulation of C_60_ in **MUV-2**, whereas the broadening and shifting of the bands towards higher frequencies are indicative of the charge-transfer (CT) interactions between the electron-acceptor C_60_ and the electron-donor TTF ligands of the framework [36,55]. On the other hand, the UV–vis spectrum of **C_60_@MUV-2** crystals dispersed in KBr pellets (Figure 3b) shows the presence of two new bands around 260 and 350 nm, which can be assigned to C_60_, whereas a broad band from 450 to 800 nm can be designated to an intermolecular CT excitation between the C_60_ and TTF ligands, as supported by theoretical calculations (see below). The experimental optical bandgap calculated from the onset is near 1.4 eV (885 nm), which is in agreement with the calculated electrochemical bandgap (1.43 eV) since the redox potential of TTF linkers was found to be 1.1 V (vs Ag/AgCl) [53] and the redox potential of C_60_ is −0.33 V (vs Ag/AgCl).

#### Gas sorption measurements

**Figure 4:**
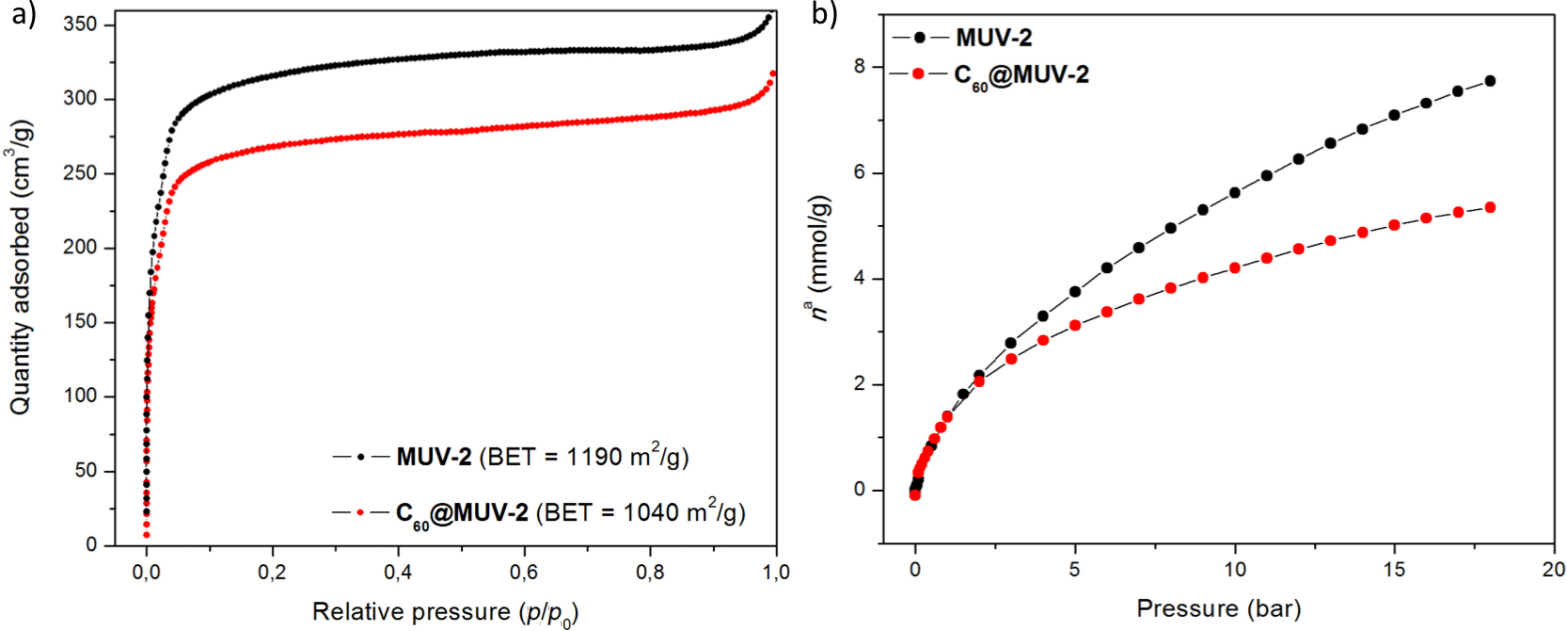
a) Nitrogen adsorption isotherms at 77 K and b) high-pressure CO_2_ adsorption isotherms at 298 K, on **MUV-2** (black) and **C_60_@MUV-2** (red).

The porosity of **C_60_@MUV-2** was studied by means of N_2_ and CO_2_ adsorption isotherm measurements (Figure 4). The measurement of nitrogen at 77 K yielded a combination of type-I and type-IV isotherms (Figure 4a), as in the case of **MUV-2**, indicating the presence of micropores and mesopores in the framework. **C_60_@MUV-2** has a Brunauer–Emmett–Teller (BET) surface area of 1040 m^2^/g, which is slightly lower than that of **MUV-2** (1190 m^2^/g). Thus, porosity is retained after encapsulation of C_60_, in agreement with other reported examples [36,56]. The pore volume decreased from 0.53 cm^3^/g to 0.44 cm^3^/g after encapsulation of C_60_ in **MUV-2**, whereas the average pore diameter calculated by means of the Barret–Joyner–Halenda (BJH) method was found to be similar in both cases (≈35 Å). The quantity of fullerene encapsulated in **MUV-2** was estimated from the decrease in pore volume, obtaining a value of around 0.7 C_60_ per 3 TTF ligands, almost 1 fullerene per section of the void. This low encapsulation rate can be explained by diffusion issues or by weak interactions between the C_60_ and the framework, which are not strong enough to keep the C_60_ retained during the washing procedure. The CO_2_ isotherm on **C_60_@MUV-2** at 298 K also showed a small decrease in the gas sorption capacity (Figure 4b), especially at high pressures (7.7 and 5.3 mmol CO_2_/g at 18 bar for **MUV-2** and **C_60_@MUV-2**, respectively).

#### Theoretical calculations

**Figure 5:**
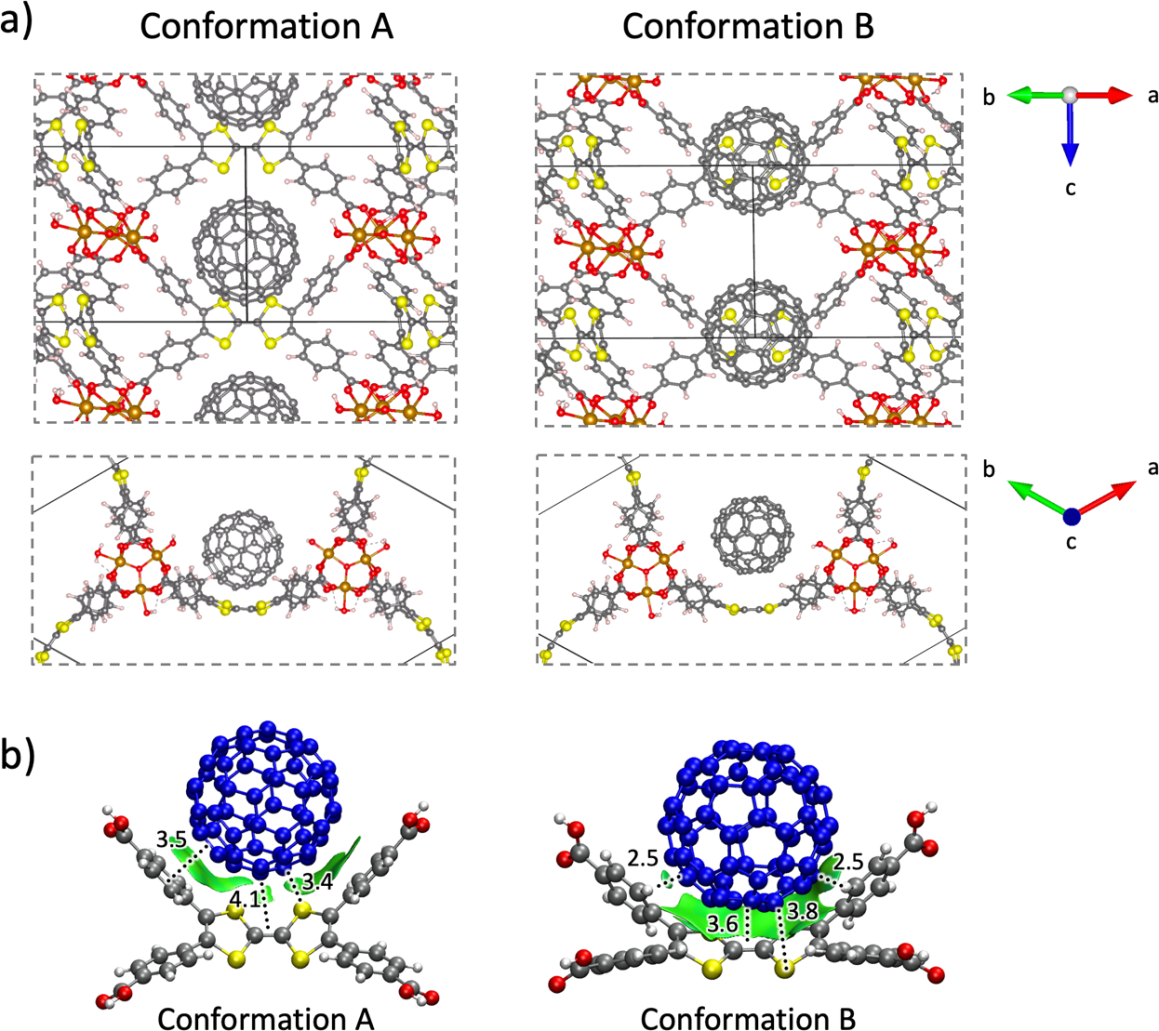
a) Minimum-energy crystal structure calculated for conformations A and B of host–guest **C_60_@MUV-2** at the PBEsol level under periodic boundary conditions (PBC). b) Supramolecular assemblies of **C_60_@TTFTB** extracted from the PBC-optimized **C_60_@MUV-2** system in arrangements A and B. Intermolecular short contacts (in Å) and NCI surfaces (reduced density gradient isovalue = 0.3 a. u. (atomic units)) are displayed. Interaction energies (*E*_int_) of −20.01 and −23.74 kcal/mol are calculated for **C_60_@TTFTB** in conformer A and B, respectively. Fullerene C_60_ is coloured in blue for better viewing.

In order to get further insight into the donor–acceptor interactions between **C_60_** and the TTF-based MOF, theoretical calculations were performed under the density functional theory (DFT). The **MUV-2** framework was modelled as previously described [53], with a high-spin Fe(III) configuration and one fullerene C_60_ guest molecule per pore (according to the experimental encapsulation efficiency). The host–guest system **C_60_@MUV-2** was fully optimized under periodic boundary conditions using the PBEsol functional with dispersion corrections (see the Experimental section for details). We initially modelled the fullerene C_60_ guest in the middle of the **MUV-2** mesopore. After several relaxation steps, the C_60_ was able to accommodate in one of the three cavities to interact favourably with the TTF-based ligand. We explored two possible conformations for the host–guest **C_60_@MUV-2** system (A and B; Figure 5a, Supporting Information File 2). In conformer A, the fullerene ball remains in the void between two TTFTB ligands, approaching one of them with short C(C_60_)^…^S(TTFTB) and C(C_60_)^…^benzene(TTFTB) contacts calculated at 3.4 and 3.5 Å, respectively (Figure 5b). In conformer B, fullerene remains over the TTFTB ligand, promoting an efficient concave–convex complementarity with a large amount of noncovalent interactions between the C_60_ ball and the TTF core (C(C_60_)^…^S(TTFTB) distances of 3.6–3.8 Å), and stabilizing CH^…^π contacts between the benzene rings of TTFTB and the fullerene (2.5 Å, Figure 5). Analysis of the NCI index allows for the visualization of the noncovalent interactions between the TTFTB ligand and the C_60_ guest, showing a significantly larger NCI surface for conformer B compared to conformer A (Figure 5b).

Accurate hybrid DFT molecular calculations including dispersion corrections were performed to quantify the total stabilization gained when C_60_ interacts with **MUV-2** in arrangements A and B. Interaction energies (*E*_int_) were calculated for the cluster **C_60_@TTFTB** at the B3LYP-D3/6-31G** level of theory with counterpoise correction, using the minimum-energy geometry previously obtained under periodic boundary conditions (see Experimental section). Theoretical calculations indicate that C_60_ favourably interacts with the TTFTB ligand, with large *E*_int_ < −20 kcal/mol in both arrangements. Conformer B, in which the fullerene is placed over the TTF moiety promoting an efficient concave–convex complementarity (Figure 5), is predicted the most stable arrangement, with an *E*_int_ value of −23.74 kcal/mol (≈4 kcal/mol more stable than conformer A). Henceforth, we focus the subsequent analysis on conformation B.

**Figure 6:**
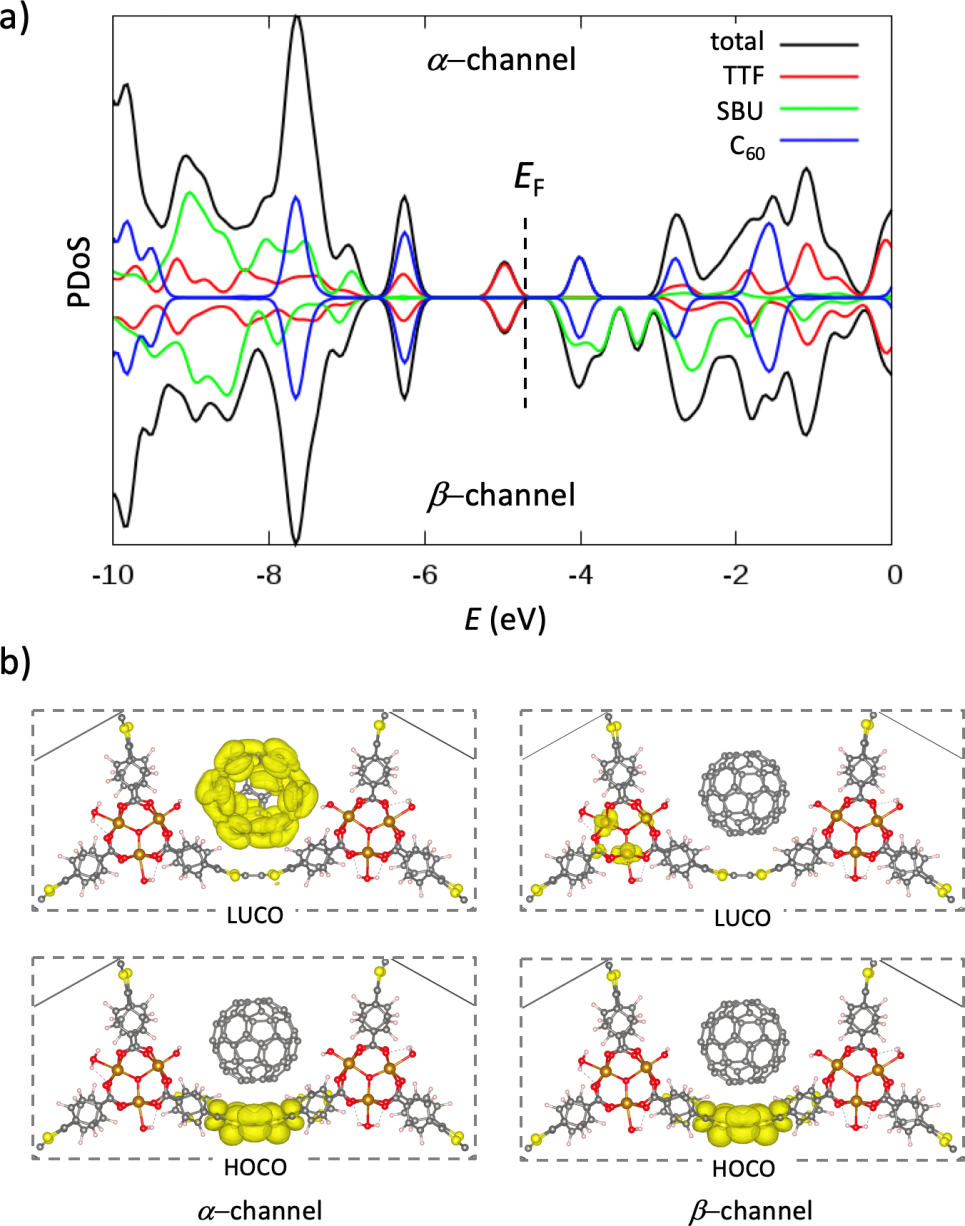
a) Projected density of states (PDoS) for the host–guest **C_60_@MUV-2** system, with contributions from the TTF core, the secondary building unit (SBU), and the fullerene C_60_. The Fermi level (*E*_F_) is indicated, and the energy reference is set to vacuum. b) Highest occupied (HOCO) and lowest unoccupied (LUCO) crystal orbitals in the two spin channels for **C_60_@MUV-2**.

Electronic structure calculations indicate that **C_60_@MUV-2** presents a small bandgap calculated to be 0.90 eV in spin-up or α-channel, and 0.72 eV in spin-down or β-channel (Figure 6), slightly smaller than that predicted for pristine **MUV-2** (0.86 eV in β-channel) [53]. Analysis of the projected density of states (PDoS) indicates that the valence band maximum (VBM) in **C_60_@MUV-2** corresponds to the electron-rich TTF unit (Figure 6a). The highest occupied crystal orbital (HOCO) displays the typical shape of the TTF HOMO and confirms the TTF-nature of the VBM (Figure 6b). In the α-channel, the conduction band minimum (CBM) is described by the fullerene moiety, being the lowest unoccupied crystal orbital (LUCO) completely localized on the C_60_ ball. Otherwise, the CBM in the β-channel is best described by the unoccupied Fe *d*-orbitals of the inorganic cluster of the MOF, the eigenstates corresponding to the fullerene being only 0.2 eV above in energy (Figure 6). Due to the relatively low bandgap, the nature of the frontier crystal orbitals and the close proximity between the electroactive donor TTF and acceptor C_60_ moieties, CT processes are expected upon light irradiation.

**Figure 7:**
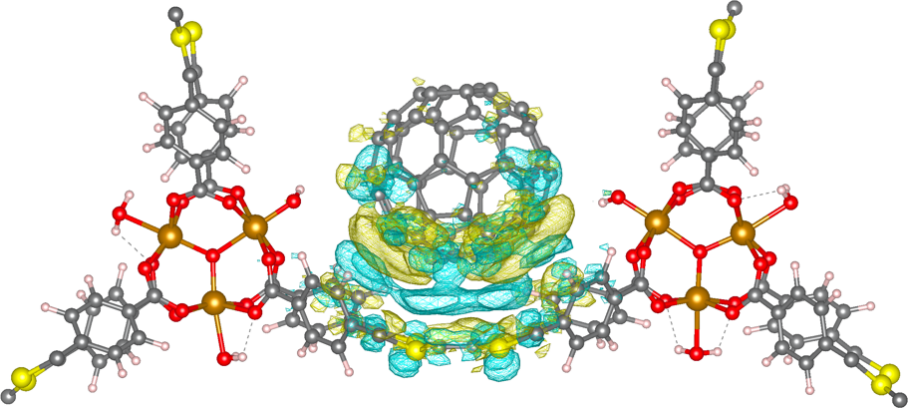
Electron density difference between host–guest **C_60_@MUV-2** and the constituting moieties (**C_60_** + **MUV-2**). Blue and yellow regions indicate charge depletion and accumulation, respectively.

Donor–acceptor interactions in **C_60_@MUV-2** were first assessed at the ground state electronic configuration. The electron density difference between the framework interacting with C_60_ (**C_60_@MUV-2**) and the individual moieties (**C_60_** + **MUV-2**) suggests a partial charge transfer from the TTF to the fullerene ball. Blue regions in Figure 7 indicate that the electron density is depleted from the TTF unit, especially from the S lone pairs, and is accumulated (yellow volumes) in the fullerene regions close to the TTFTB ligand. The partial charge transfer from the donor TTFTB ligand to the acceptor fullerene moiety in the ground state is calculated to be as small as 0.02e (and probably assigned to electronic polarization), with an exponential decay as a function of the C_60_^…^TTF intermolecular distance (Table S2, Supporting Information File 1).

**Figure 8:**
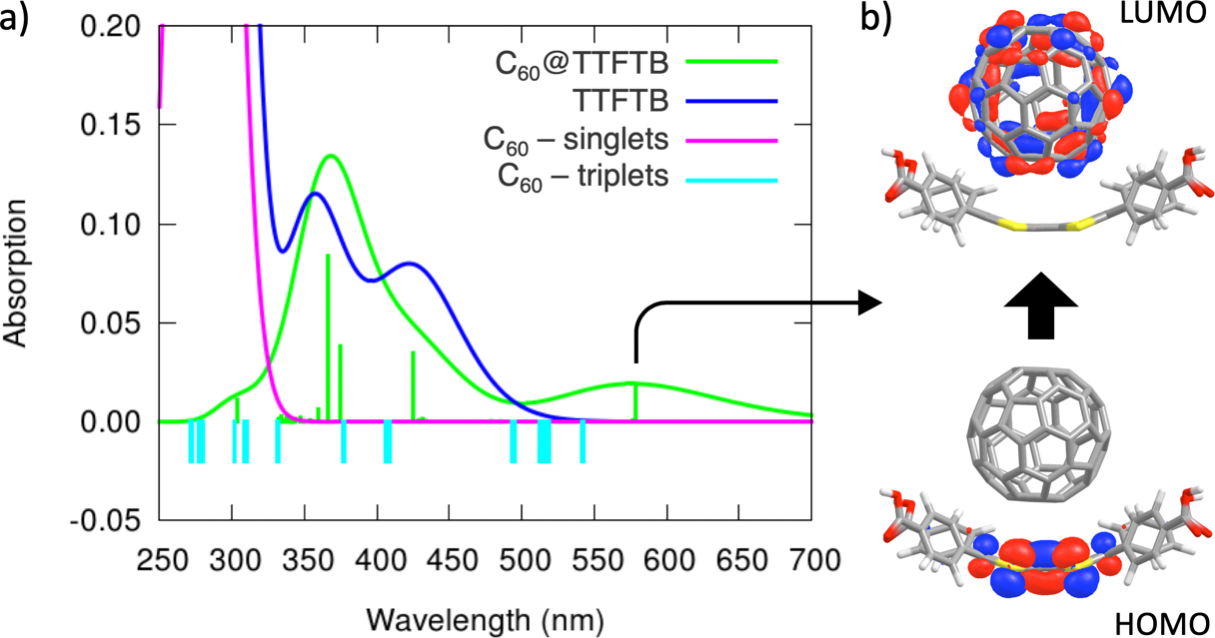
a) TDDFT absorption spectra calculated at the CAM-B3LYP/6-31G** level for host–guest **C_60_@TTFTB** (including vertical excitation energies), TTFTB ligand, and fullerene C_60_ (singlets and triplet energies). b) Monoelectronic excitation that describes the nature of the lowest-lying singlet excited state S_1_ of **C_60_@TTFTB**.

Time-dependent DFT molecular calculations were performed at the CAM-B3LYP/6-31G** level (see the Experimental section) to shed light onto the photoinduced CT process of **C_60_@MUV-2**. Figure 8 displays the simulated absorption spectra predicted for the cluster **C_60_@TTFTB** system in the most stable arrangement B, the TTFTB ligand, and the fullerene guest (the triplet excitation energies are indicated). The high-energy region (below 400 nm) of the experimental UV–vis absorption spectrum of **C_60_@MUV-2** is dominated by the **MUV-2** framework (Figure 3b). Theoretical calculations predict several intense transitions in the region below 300 nm for the TTFTB ligand (Table S3, Supporting Information File 1) that explain the experimental wide band with maximum at ≈230 nm recorded for **MUV-2** and **C_60_@MUV-2**. These transitions are described by π–π* electronic promotions involving the TTF and the peripheral carboxybenzene groups in the TTFTB ligand (Table S3, Supporting Information File 1). Singlet excited states S_1_ and S_3_ are predicted with less intensity (oscillator strength *f* < 0.2) and are described by TTF→benzene and TTF-centred monoexcitations, respectively, and give rise to the shoulder experimentally recorded at ≈350 nm for **C_60_@MUV-2** (Table S3, Supporting Information File 1). On the other hand, the predicted singlet excited states of fullerene S_37_–S_39_ (*f* ≈ 0.2) and S_52_–S_54_ (*f* ≈ 0.1) in the region of 280 and 260 nm (Table S3, Supporting Information File 1), respectively, correlate with the experimental features that appear at 325 and 275 nm in host–guest **C_60_@MUV-2** (Figure 3b).

Importantly, a new singlet excitation is computed for **C_60_@TTFTB** (S_1_) at 578 nm, which is not predicted either for the TTFTB ligand or the fullerene C_60_ (Figure 8). This transition has relatively small intensity (*f* = 0.018), and can be described by one-electron promotion from TTF to C_60_, i.e., it has a CT nature (Figure 8b). The position of this CT excitation (578 nm) nicely agrees with the low-energy broad band that appears in the experimental absorption spectrum of **C_60_@MUV-2,** centred at 550 nm and expanding up to 800 nm. In fact, theoretical calculations indicate that the intensity and energy of the S_1_ CT transition in **C_60_@TTFTB** is significantly affected by the intermolecular TTF^…^C_60_ distance and the characteristic TTF boat dihedral angle (Table S4, Supporting Information File 1). The charge transfer from the TTF to the fullerene ball in the S_1_ CT excitation of **C_60_@TTFTB** is calculated to be of nearly 1e (0.94e at the minimum-energy geometry, Table S2, Supporting Information File 1).

#### Electrical measurements

**Figure 9:**
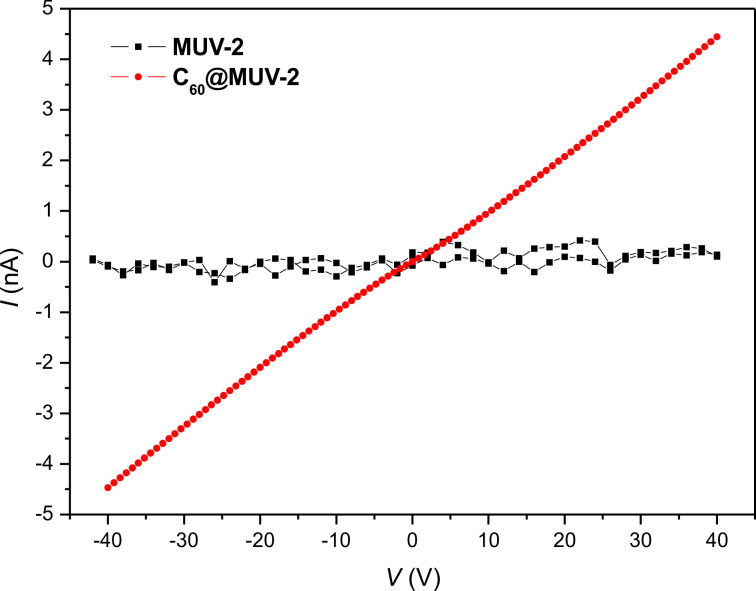
Current (*I*)–Voltage (*V*) plot for pressed pellets of **MUV-2** (black) and **C_60_@MUV-2** (red) at 300 K.

In order to analyse the possible enhancement of electrical conductivity after encapsulation of C_60_ in **MUV-2**, transport measurements for **MUV-2** and **C_60_@MUV-2** were performed using two-contact probe pressed-pellet devices measured at room temperature (300 K) (Figure 9). Interestingly, the pellet of **C_60_@MUV-2** shows an increase of around two orders of magnitude (σ = 4.7·10^−9^ S/cm) compared to the very resistive **MUV-2** (σ = 3.7·10^−11^ S/cm, Table 1). This enhancement of the electrical conductivity can be explained by the donor–acceptor charge transfer from the TTF linkers to C_60_ since the fullerene is acting as a dopant introducing charge carriers within the framework. However, this enhancement in conductivity is lower in comparison to other reported systems [14,35] probably due to the low ratio between C_60_ and TTF (1:4) and the long distances between the TTF moieties (9.6 Å along the *c*-axis), which could prevent the charge delocalisation along the framework.

**Table 1:** Geometrical factors (length *l*, width *w*, and thickness *t*), resistance obtained by the linear fit of the ohmic regime of the *I*–*V* curves and conductivity for **MUV-2** and **C_60_@MUV-2**, at 300 K.

sample	*l* (μm)	*w* (μm)	*t* (μm)	*R* (Ω) at 300 K	σ (S/cm) at 300 K

**MUV-2**	110	480	65	(9.5 ± 0.6)·10^11^	3.7·10^−11^
**C_60_@MUV-2**	325	540	150	(8.510 ± 0.015)·10^9^	4.7·10^−9^

## Conclusion

In summary, we have reported for the first time the encapsulation of C_60_ in a mesoporous TTF-based MOF (**MUV-2**). Charge-transfer interactions between C_60_ and TTF ligands from the framework in **C_60_@MUV-2** were confirmed by different spectroscopic techniques and theoretical calculations. Interestingly, after encapsulation of fullerenes, gas sorption measurements demonstrated that the mesoporosity of the MOF is maintained, and electrical measurements revealed an increase of around two orders of magnitude in conductivity, which can be explained by CT donor–acceptor (TTF→C_60_) interactions. Current research is focused on the improvement of the electrical conductivity in **MUV-2** and the photophysical characterisation of the charge transfer process in **C_60_@MUV-2**.

## Experimental

**General methods and materials:** All reagents and solvents employed for the syntheses were of high purity and were purchased from Sigma-Aldrich Co., and TCI. Powder X-ray diffraction patterns were recorded using 0.7 mm borosilicate capillaries that were aligned on an Empyrean PANalytical powder diffractometer, using Cu Kα radiation (λ = 1.54056 Å). Raman spectra were acquired with a micro-Raman (model XploRA ONE from Horiba, Kyoto, Japan) with a grating of 1200 gr/mm and a wavelength of 785 nm. UV–vis absorption spectra were recorded on a Jasco V-670 spectrophotometer in baseline mode from 400 to 800 nm range. The absorption spectra were measured on the solid state by dispersing the crystals in KBr pellets. Nitrogen adsorption isotherms were measured using a TriStar II PLUS apparatus (Micromeritics) at 77 K. The BET surface area was calculated by using the Brunauer–Emmett–Teller equation. The high-pressure CO_2_ adsorption isotherms were measured in a IGA-100 gravimetric sorption analyzer (Hiden Isochema) and the sample was degassed for 2 h at 150 °C in vacuum.

**Synthesis of MUV-2 and encapsulation of C_60_: MUV-2** was synthesised and characterised as previously reported [52]. Then, 30 mg of fullerene (C_60_) was dissolved in 2 mL of *o*-dichlorobenzene and activated **MUV-2** (10 mg) was added to it. The vial was heated at 60 °C for 3 days and the MOF was then exhaustively washed with *o*-dichlorobenzene to remove any physisorbed C_60_ on the MOF surface, washed with methanol and finally dried at 150 °C for 2 h.

**Computational details:** Theoretical calculations were performed under the density functional theory framework. Periodic boundary conditions (PBC) calculations were carried out with the FHI-AIMS (Version 171221) software [57]. **MUV-2** was modelled as previously described, with Fe(III) ions in a high-spin d^5^-configuration. The guest C_60_ molecule was rationally inserted into the bigger mesoporous channel of **MUV-2** in the most plausible sites, and the geometry of the host–guest **C_60_@MUV-2** system was fully relaxed at the PBEsol functional [58] with tier-1 basis set. Dispersion corrections were added according to the Hirshfeld partitioning of the electron density (Tkatchenko–Scheffler method) [59]. Electronic structure calculations were performed for band structure analysis using the hybrid HSE06 functional [60] and tier-1 basis set. Energy reference was set to vacuum according to the protocol reported by Butler and co-workers [61]. Crystal structures, crystal orbitals and electron density differences were plotted by means of VESTA (version 3.4.6) software [62]. NonCovalent Index (NCI) calculations were performed under the NCIPLOT-3.0 software [63–64] using the default PROMOLECULAR atomic densities, and density and gradient thresholds. The intermolecular contribution to the NCI surfaces was calculated by means of the INTERMOLECULAR keyword, and the VMD-1.9.3 software [65] was employed for graphical display. Molecular DFT calculations were performed for the **C_60_@TTFTB** system using the Gaussian-16.A03 suite of packages [66]. Hydrogen atoms were added in the terminal carboxylate groups for charge neutrality. Interaction energies were calculated for the previously PBC-optimized crystal structures as the energy difference between the dimer and the constituting monomers. The hybrid B3LYP [67] with the Grimme’s D3 dispersion correction [68] (B3LYP-D3) was employed along with the 6-31G** basis set and half of the counterpoise correction (CP) [69]. The consistency of the interaction energy trends at the B3LYP-D3/6-31G**+½CP level was confirmed by using other basis set or CP weights (Table S1, Supporting Information File 1). Time-dependent DFT (TDDFT) calculations were performed using the coulomb-attenuating CAM-B3LYP approach [70] with the 6-31G** basis set for the lowest-lying excited states. The B3LYP functional was also tested, but the characteristic charge-transfer excitation was largely underestimated (Figure S4, Supporting Information File 1). Excitation energies were convoluted with Gaussian functions with full-width-at-half-maximum (FWHM) of 0.2 eV. Charge transfer was evaluated as the accumulated natural population analysis (NPA) [71] charges on each moiety. Molecular orbitals were represented by means of the Chemcraft 1.7 software [72].

**Electrical measurements:** Pressed pellets (*F* ≈ 5 US tons) were cut in rectangular shapes and contacted with silver conductive paint (RS 123-9911) and platinum wires (Goodfellow, 99.99%, 25 μm of diameter) in a four-probe configuration (Figure S2 and Figure S3, Supporting Information File 1). The geometrical factors (thickness, width and length were measured using an optical microscope (width and length were determined from the top view, Figure S2a and Figure S3a, and the thickness from the lateral view, Figure S2b and Figure S3b). *I*–*V* curves were measured with a Keithley 6517B electrometer for ultra-high resistance/ultra-low current measurements in a two-probe configuration, i.e., applying a voltage bias between two leads and measuring the current between them.

## Supporting Information

File 1 Additional figures and tables.

File 2 CIF files of simulated structures of C_60_@MUV-2.
The ZIP archive contains CIF files of the simulated structures of C60@MUV-2 in conformation A and in conformation B.
