# Supplementary material for: Charge-transfer interactions between fullerenes and a mesoporous tetrathiafulvalene-based metal–organic framework
Source: Beilstein J Nanotechnol. 2019 Sep 18;10:1883–93. doi: 10.3762/bjnano.10.183 (PMC6774073; doi:10.3762/bjnano.10.183)
Supplement: File 1 — Additional figures and tables. [file Beilstein_J_Nanotechnol-10-1883-s001.pdf]

BEILSTEIN JOURNAL OF NANOTECHNOLOGY

Supporting Information

for

Charge-transfer interactions between fullerenes and a mesoporous tetrathiafulvalene-based metal–organic framework

Manuel Souto, Joaquín Calbo, Samuel Mañas-Valero, Aron Walsh and Guillermo Mínguez Espallargas

Beilstein J. Nanotechnol. **2019,** *10,* 1883–1893. doi:10.3762/bjnano.10.183

Additional figures and tables

License and Terms: This is a supporting information file under the terms of the Creative Commons Attribution License (http://creativecommons.org/licenses/by/4.0). Please note that the reuse, redistribution and reproduction in particular requires that the authors and source are credited. The license is subject to the *Beilstein Journal of Nanotechnology* terms and conditions: (https://www.beilstein-journals.org/bjnano)

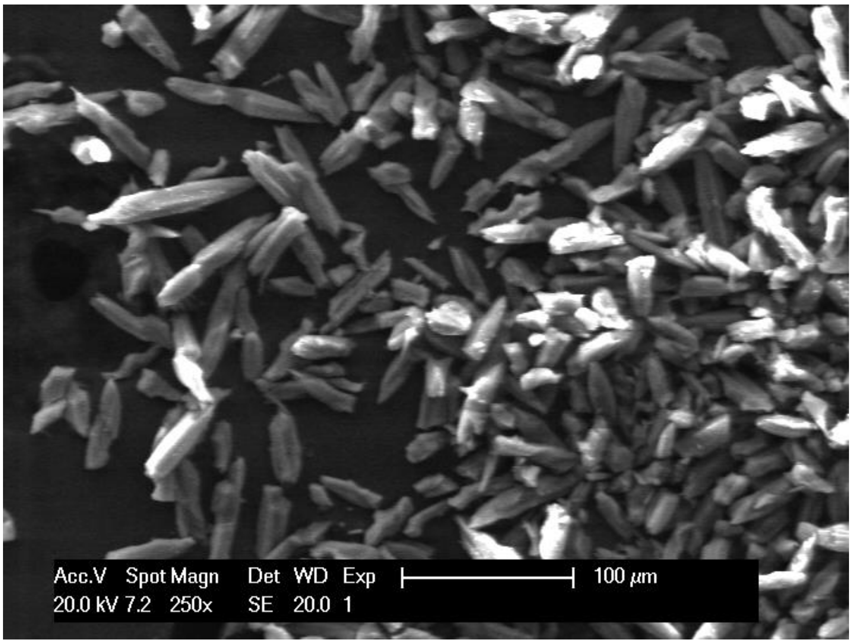

Figure S1: SEM image of **C_{60}@MUV-2**.

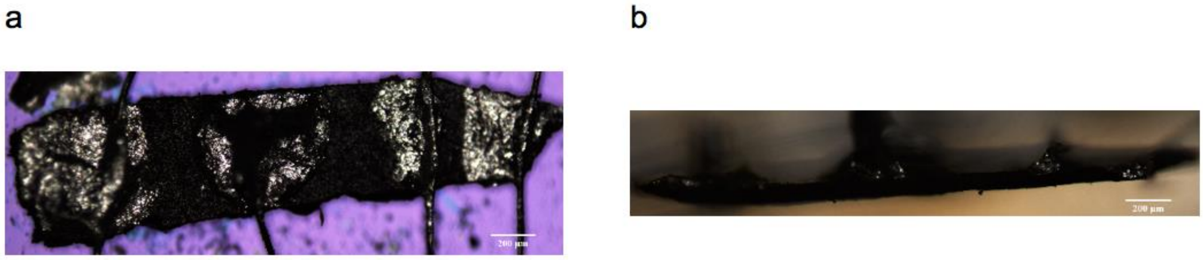

Figure S2: Top view (a) and lateral view (b) for **MUV-2** electrical measurements**.**

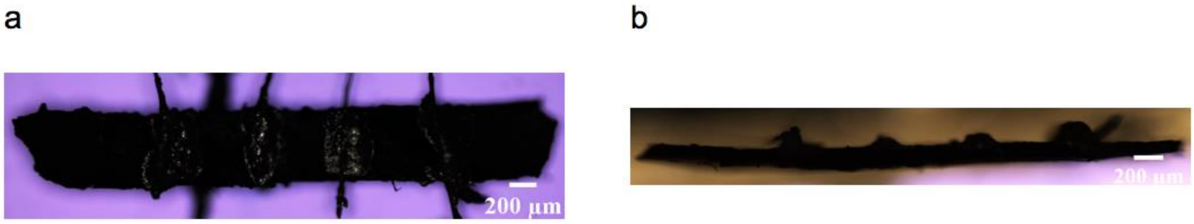

Figure S3: Top view (a) and lateral view (b) for **C_{60}@MUV-2** electrical measurements**.**

Table S1: Interaction energy (in kcal/mol) of the two conformations A and B of the host-guest **C_{60}@MUV-2** complex at the B3LYP level using different basis sets and including or not the counterpoise (CP) correction.

	**Conformation A**		**Conformation B**	
	6-31G**	6-31+G*	6-31G**	6-31+G*
**No CP**	-23.00	-22.10	-26.85	-24.96
**½ CP**	-20.01	-19.99	-23.72	-22.86
**Full CP**	-17.02	-17.88	-20.59	-20.75

Table S2: Charge transfer (in electron charge units, e) from the electron-donor TTFTB ligand to the electron-acceptor C_{60} guest in the ground state (GS) and the charge-transfer S_1 state (CT) of **C_{60}@TTFTB** as a function of the distance between centroids (*d*). Data corresponding to the minimum-energy geometry is in bold.

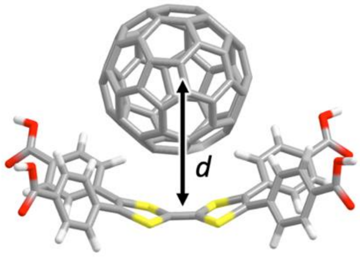

d **(Å)**	**GS**	**CT**
6.61	0.05	0.92
**6.86**	**0.02**	**0.94**
7.11	0.01	0.96
7.36	0.00	0.97
7.61	0.00	0.98
7.86	0.00	0.99
8.11	0.00	0.99

Table S3: Intense lowest-lying singlet excited states of TTFTB ligand and C_{60} guest, calculated at the CAM-B3LYP/6-31G** level of theory. The energy (*E*), oscillator strength (*f*), major monoexcitation, and description of each state are included.

State	*E* (eV)	*E* (nm)	*f*	Monoexcitation	Description
			TTFTB		
S_1	2.907	426.4	0.0760	H → L (68%)	TTF → peripheral
S_3	3.486	355.7	0.1043	H → L+4 (58%)	TTF
S_6	4.261	291.0	0.4840	H → L+3 (52%)	TTF → peripheral
S_8	4.430	279.9	0.5490	H-1 → L+1 (51%)	TTF → peripheral
S_{21}	5.217	237.7	0.4389	H-3 → L (15%)	TTF → peripheral
S_{54}	6.623	187.2	0.7300	H-4 → L+5 (13%)	TTF + peripheral
			C_{60}		
S_{37}	4.309	287.7	0.2196	H-2 → L+3 (18%)	-
S_{38}	4.312	287.6	0.2196	H-3 → L+5 (21%)	-
S_{39}	4.315	287.3	0.2206	H-4 → L+5 (31%)	-
S_{52}	4.703	263.7	0.1192	H-6 → L+2 (19%)	-
S_{53}	4.704	263.6	0.1181	H-6 → L+1 (21%)	-
S_{54}	4.706	263.5	0.1133	H-8 → L (17%)	-

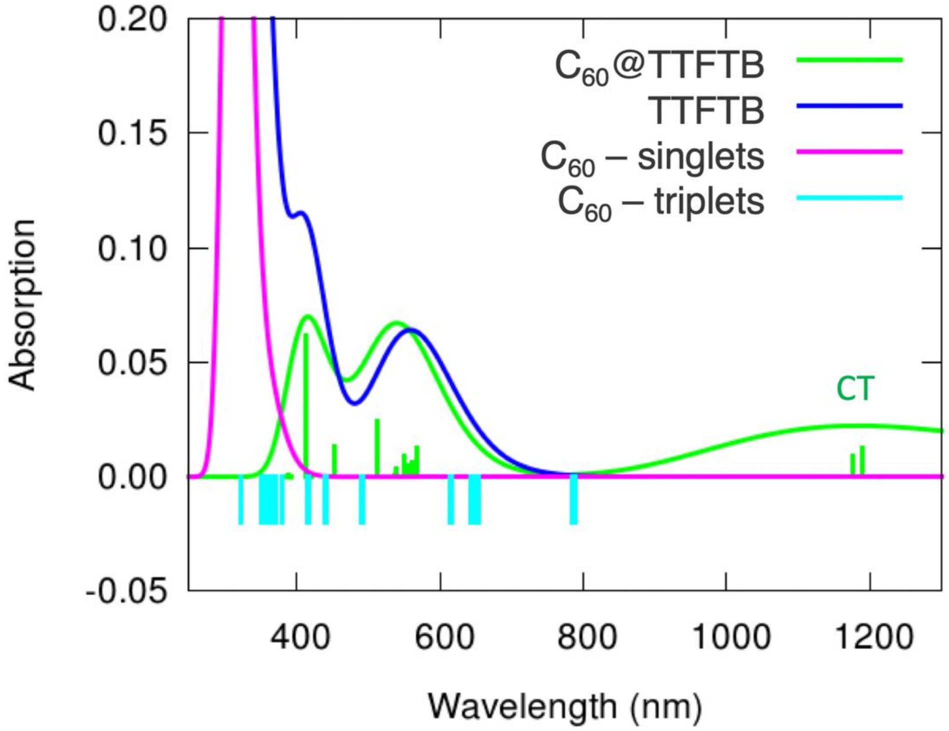

Figure S4: TDDFT absorption spectra calculated at the B3LYP/6-31G** for host-guest **C_{60}@TTFTB (**including vertical excitation energies), TTFTB ligand, and fullerene C_{60} (singlets and triplet energies).

Table S4: Evolution of S_1 CT excitation for **C_{60}@TTFTB** as a function of distance *d* (as defined in Table S1) and the TTF boat dihedral angle (θ). Data corresponding to the minimum-energy geometry is in bold.

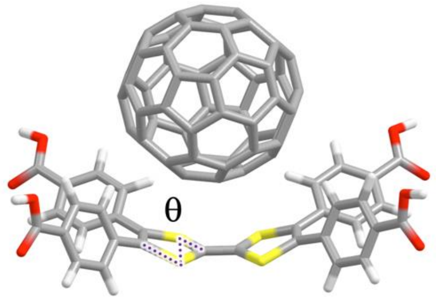

d (Å)	*E* (eV)	*E* (nm)	*f*
6.61	2.146	577.8	0.0284
**6.86**	**2.143**	**578.6**	**0.0178**
7.11	2.167	572.1	0.0103
7.36	2.209	561.3	0.0054
7.61	2.258	549.0	0.0027
7.86	2.310	536.8	0.0012
8.11	2.360	525.4	0.0005
**θ (º)**	***E* (eV)**	***E* (nm)**	***f***
**162**	**2.143**	**578.6**	**0.0178**
171	2.044	606.7	0.0131
180	2.025	612.3	0.0079
